# Upper airway obstruction during extubation after general anesthesia, in a patient with Parkinson disease: A case report

**DOI:** 10.1097/MD.0000000000020363

**Published:** 2020-05-22

**Authors:** Xiaodi Sun, Leyang Dai, Yinbing Pan, Huanhuan Sha

**Affiliations:** Department of Anesthesiology and Perioperative Medicine, The First Affiliated Hospital of Nanjing Medical University, Nanjing Jiangsu, P.R. China.

**Keywords:** bilateral vocal cord paralysis, extubation, general anesthesia, Parkinson disease, upper airway obstruction

## Abstract

**Rationale:**

Parkinson disease (PD) is a chronic neurodegenerative condition often suffered by the elderly. Upper airway obstruction, though rare in patients with PD, can be life threatening and is associated with vocal cord paralysis, laryngeal spasm, and dystonia of jaw and neck muscles.

**Patient concerns:**

We describe a life-threatening upper airway obstruction caused by bilateral vocal cord paralysis, in an elderly man with PD, during extubation after general anesthesia.

**Diagnoses:**

Based on clinical presentation and visual laryngoscopy, the patient was diagnosed with laryngeal spasm and bilateral vocal cord paralysis after extubation.

**Interventions:**

Re-intubation was carried out and dopamine hydrazine tablets were administered via a nasal feeding tube.

**Outcomes:**

After re-intubation and further treatment, the endotracheal tube was successfully removed and no symptoms of respiratory distress were observed.

**Lessons:**

Patients with PD may be at a risk of life-threatening upper airway obstruction after extubation, which should be prevented systematically.

## 1 Introduction

Parkinson disease (PD) is a chronic, neurodegenerative disorder that affects the extrapyramidal system. Apart from limited motor functions such as rigidity, rest tremor, and slowness of movement, PD can affect pharyngeal sensory nerves and impair swallowing and airway protective reflexes, as manifested by dysphagia, secretion retention, and aspiration.^[1,2]^ Respiratory dysfunction has been noted in PD and is attributed to impaired central control of respiratory muscles, upper airway obstruction, and laryngeal muscle atony. A rare complication of PD is vocal cord paralysis, presenting as hoarseness, dysphonia, dyspnea, and reflux aspiration.^[3]^ Liu^[4]^ has reported a case of persistent perioperative laryngeal spasm in a patient with PD. Another case report describes a 71-year-old man with PD, who developed upper airway obstruction requiring intubation, after the discontinuation of his Parkinson medication.^[5]^ In this case report we highlight the rare complication of severe upper airway obstruction caused by bilateral vocal cord paralysis in a patient with PD, that occurred during extubation after general anesthesia, likely due to the withdrawal of dopaminergic medication.

## 2 Case presentation

A 72-year-old man (158 cm, 67 kg) was scheduled for transurethral prostatic balloon dilation, under general anesthesia. He had a 2-year history of PD, and managed the symptoms by taking dopamine hydrazine tablets regularly. The patient had no history of hypertension, diabetes, asthma, smoking, or drinking. The results of routine laboratory examinations were normal. Echocardiography suggested decreased left ventricular diastolic function and mild mitral valve insufficiency. Examination of pulmonary function suggested a mild restrictive ventilatory dysfunction. At 07:00 on the day of surgery, 250 mg dopamine hydrazine tablet was administered to the patient. He was transferred to the operating room at 11:45. Electrocardiogram monitoring showed a heart rate of 72 beats/min, a radial blood pressure of 170/82 mm Hg and oxygen saturation of 96%. General anesthesia was induced by intravenous administration of anesthetics and was maintained with a combination of intravenous and inhaled anesthetics. The procedure lasted 30 minutes, and the patient was transferred to the post-anesthesia care unit at 13:10. Thirty minutes later, the patient was awake and able to obey verbal commands (with head raised up for more than 5 seconds, and a firm handshake), so the endotracheal tube was removed. The patient immediately presented shortness of breath accompanied by wheezing, and obvious 3 concave signs, and the oxygen saturation gradually dropped to 89%. We immediately supplied oxygen via facemask, but the airway resistance was relatively high and the assisted breathing was not satisfactory. The symptoms were not relieved. Propofol was administered, secretions were removed under visual laryngoscopy and 2% lidocaine was sprayed at the glottis. The wheezing stopped when the patient stopped breathing, and reappeared when the patient breathed spontaneously once again. We observed by laryngoscope, that the patient's glottis closed on inhalation and opened on exhalation. After repeating the treatment as before, there was no improvement in the symptoms, so the trachea was re-intubated. One hour later the patient was conscious, and the endotracheal tube was removed, the upper airway tract obstruction returned. The otolaryngologist suspected that the patient had a vocal cord paralysis, and suggested a tracheotomy, but the patient's family refused. Therefore, the trachea was re-intubated again. Based on a comprehensive analysis of the patient's medical history, it was considered that the refractory upper airway obstruction after extubation, might be related to discontinuation of the patient's PD medication. Therefore, 250 mg dopamine hydrazine tablets were given through the nasal feeding tube at 19:00. One hour later, the endotracheal tube was removed, and the symptoms of upper airway obstruction were improved. The patient was awake and breathing smoothly. The arterial blood gas showed a pH of 7.39, partial pressure of oxygen of 96 mm Hg and partial pressure of carbon dioxide of 44 mm Hg, with the patient breathing oxygen via a nasal cannula. Dopamine hydrazine tablets 250 mg were administrated again at 20:20. Further investigation of the patient's history revealed that, 1 year after the diagnosis of PD, he was admitted to the department of otolaryngology due to difficulty in speaking and mild hoarseness. The patient was diagnosed with bilateral vocal cord paralysis by laryngoscopy but received no treatment as the symptoms were mild.

## 3 Discussion

Upper airway tract obstruction is not common in patients with PD, but can be life threatening, associated with laryngeal spasm, vocal cord paralysis, and dystonia of jaw and neck muscles.^[6,7]^ In our patient, life-threatening dyspnea occurred because of upper airway obstruction caused by persistent laryngeal spasm, which developed during extubation after general anesthesia.

Laryngeal spasm refers to involuntary contraction of the laryngeal muscles, vocal cord adduction and partial or complete closure of the glottis. It causes different degrees of airway difficulties, including complete airway obstruction. Sputum suction or residual muscle relaxation may induce laryngeal spasm. Mild or moderate laryngeal spasm may manifest as incomplete upper airway obstruction, accompanied by a laryngeal purr, and can be relieved by positive-pressure facemask ventilation, under sedation with propofol. Severe laryngeal spasm can result in dyspnea, and the patient may need emergency endotracheal intubation. Laryngeal spasm is not difficult to manage generally, however, in the present case the upper airway obstruction was not relieved when the patient was treated with suitable therapies. Considering our patient's medical history and the result of video laryngoscopy, we concluded that vocal cord paralysis rather than possible residual muscle relaxation after surgery was responsible for the persistent laryngeal spasm. In this case, vocal cord paralysis was present before surgery according to the patient's history, but there were no obvious symptoms of choking cough, wheezing, or dyspnea, which suggests that the vocal cord paralysis in this patient was mild.

It has been shown that persistent over activity of the intrinsic laryngeal muscles (extrapyramidal involuntary movements) is associated with vocal fold paralysis in PD.^[8]^ Several authors have suggested that the mechanism responsible for vocal cord paralysis involves dystonia and spasm of the adductor laryngeal muscles.^[6,9,10]^ Drug therapy is the main treatment for PD, and levodopa or dopamine receptor agonists are the cornerstone of the treatment.^[11]^ The continuation of anti-parkinsonian drugs throughout the perioperative period is essential, as they reduce abnormal function of the upper airway. In Liu's case,^[4]^ dopaminergic medication was resumed and the symptoms completely resolved. von Eckardstein^[12]^ also reported a case of acute drug withdrawal causing airway obstruction, which suggested that inadequate drug treatment may result in persistent laryngeal spasm and adjusting the drug dose can alleviate the symptoms in PD patients. We hypothesize that the severe bilateral vocal cord paralysis was due to the withdrawal of dopaminergic medication in our patient. However, von Eckardstein^[12]^ has stated that 12 hours of levodopa withdrawal during preoperative evaluation, was not likely to be long enough to result in a clinically relevant laryngeal spasm. He proposed to allow levodopa medication up to 6 hours before surgery. In our case, the patient was given 250 mg hydrazine tablets at 07:00 on the day of surgery. At the time of extubation, it was 7 hours since the last administration, so the withdrawal time of the anti-parkinsonian drugs was not very long. In this case, after the administration of 250 mg dopamine hydrazine tablets at 19:00, and again at 20:20, the symptoms of upper airway obstruction gradually improved. When transferred to the ward, the patient continued his regular medication regimen and the symptoms of dyspnea and wheezing were significantly reduced by the next day. This suggests that the postoperative upper airway obstruction, related to bilateral vocal cord paralysis, was caused by acute reduction of drug concentration. On the other hand, we thought that general anesthesia also played a role in the vocal cord paralysis. Inhalational anesthetics have complex effects on brain dopamine concentrations during general anesthesia. Opioids inhibit dopamine release in the central nervous system and increase the likelihood of muscle rigidity. So, we concluded that the acute withdrawal of anti-parkinsonian drugs was responsible for the vocal cord paralysis, exacerbated by the effects of general anesthesia and surgery.

The following points should be borne in mind, regarding the perioperative use of anti-parkinsonian drugs:

(1) The withdrawal time should not be long because of the short half-life of levodopa. It should be administered in the morning, before surgery, and as soon as possible after surgery.
(2) Common adverse reactions to levodopa or dopamine agonists include hypotension, nausea and vomiting, and abdominal discomfort, which may cause dehydration or insufficient blood volume.
(3) During prolonged surgery, patients could suffer from muscle rigidity because of the short half-life of levodopa. Drug administration via a nasal feeding tube can prevent the onset of Parkinson symptoms in such a case.

In summary, anesthetists must consider the possibility of upper airway tract obstruction during the perioperative period, for PD patients requiring general anesthesia with endotracheal intubation. The evaluation of hoarseness, choking cough, and dyspnea should be performed carefully, prior to surgery. These symptoms may suggest bilateral vocal cord paralysis, which might lead to refractory and life-threatening upper airway obstruction after extubation. General anesthesia should be approached cautiously, in patients with vocal fold paralysis. Because of preoperative fasting, drugs should be available, via a nasal feeding tube, in time to prevent the onset of symptoms.

## Acknowledgments

We would like to thank professor Zhengnian Ding and Cunming Liu for the rescue of this patient.

## Author contributions

All authors have made material contributions to this manuscript according to the rules of authorship as explained in the ICMJE guidelines., DLY, PYB and SHH were the anesthetists of this patient, DLY contributed to the consent form of the patient. SXD and SHH were major contributors in writing the manuscript and all authors read and approved the final manuscript.

**Conceptualization:** Xiaodi Sun, Yinbing Pan, Huanhuan Sha.

**Formal analysis:** Xiaodi Sun, Leyang Dai, Huanhuan Sha.

**Writing – original draft:** Xiaodi Sun, Huanhuan Sha.

**Writing – review & editing:** Xiaodi Sun, Huanhuan Sha.

## References

[1] Sveinbjornsdottir S The clinical symptoms of Parkinson's disease. J Neurochem 2016;139: Suppl 1: 318–24. 27401947 10.1111/jnc.13691

[2] Nicholson G Pereira AC Hall GM Parkinson's disease and anaesthesia. Br J Anaesth 2002;89:904–16. 12453936 10.1093/bja/aef268

[3] Seyed Toutounchi SJ Eydi M Golzari SE Vocal cord paralysis and its etiologies: a prospective study. J Cardiovas Thorac Res 2014;6:47–50. 10.5681/jcvtr.2014.009 PMC3992732 24753832

[4] Liu EJ Dhara S Stubborn perioperative laryngeal spasm in a patient with Parkinson disease. Can J Anaesth 1998;45:495–7. 10.1007/BF03012589 9598268

[5] Easdown LJ Tessler MJ Minuk J Upper airway involvement in Parkinson's disease resulting in postoperative respiratory failure. Can J Anaesth 1995;42:344–7. 7788833 10.1007/BF03010713

[6] Torsney KM Forsyth D Respiratory dysfunction in Parkinson's disease. J R Coll Physicians Edinb 2017;47:35–9. 28569280 10.4997/JRCPE.2017.108

[7] Gan EC Lau DP Cheah KL Stridor in Parkinsonʼs disease: a case of ʻdry drowningʼ? J Laryngol Otol 2010;124:668–73. 20003593 10.1017/S0022215109992222

[8] Qayyum A Mierzwa K See M Laser arytenoidectomy for bilateral vocal fold palsy in Parkinsonʼs disease. J Laryngol Otol 2005;119:831–3. 16259666 10.1258/002221505774481345

[9] Marion MH Klap P Perrin A Stridor and focal laryngeal dystonia. Lancet 1992;339:457–8. 1346820 10.1016/0140-6736(92)91060-l

[10] Blitzer A Brin MF Laryngeal dystonia: a series with botulinum toxin therapy. Ann Otol Rhinol Laryngol 1991;100:85–9. 1992905 10.1177/000348949110000201

[11] Kalia LV Lang AE Parkinsonʼs disease. Lancet 2015;386:896–912. 25904081 10.1016/S0140-6736(14)61393-3

[12] von Eckardstein KL Sixel-doring F Kazmaier S Asphyxia due to laryngeal spasm as a severe complication of awake deep brain stimulation for Parkinsonʼs disease: a case report. BMC Neurol 2016;16:216–9. 27821134 10.1186/s12883-016-0736-7 PMC5100222

